# Supplementary material for: Monitoring trends and differences in COVID-19 case-fatality rates using decomposition methods: Contributions of age structure and age-specific fatality
Source: PLoS One. 2020 Sep 10;15(9):e0238904. doi: 10.1371/journal.pone.0238904 (PMC7482960; doi:10.1371/journal.pone.0238904)
Supplement: S1 File — (DOCX) [file pone.0238904.s001.docx]

**Monitoring trends and differences in COVID-19 case fatality rates using decomposition methods: Contributions of age structure and age-specific fatality**

Christian Dudel, Tim Riffe, Enrique Acosta, Alyson van Raalte, Cosmo Strozza, Mikko Myrskylä

Corresponding author:

Christian Dudel

Max Planck Institute for Demographic Research

Konrad-Zuse-Str. 1

18057 Rostock

Germany

Email: dudel@demogr.mpg.de

# A. Details of the decomposition method

## A.1 Decomposing CFRs

We want to decompose, or “explain”, the difference between two CFRs, irrespective of whether they are from two different populations, or from the same population at two different points in time, or from different groups within a population, e.g., gender or socio-economic groups. We will use ${CFR}_{i}$ and ${CFR}_{j}$ to distinguish the two CFRs, e.g., country $i$ and country $j$. Moreover, we write $P_{ia}$, $C_{ia}$, $P_{ja}$, and $C_{ja}$ for the underlying age compositions and age-specific CFRs; i.e., ${CFR}_{i}=\sum P_{ia}C_{ia}$.

Using a decomposition approach introduced by Kitagawa [1] we separate the difference between two CFRs into two distinct parts,

$${CFR}_{i}-{CFR}_{j}=\alpha+\delta,$$

where $\alpha$ captures the part of the difference between CFRs which is due to differences in the age composition of cases, and $\delta$ is due to differences in mortality. $\alpha$ is given by

$$\alpha=0.5\left( {CFR}_{i}-\sum P_{ja}C_{ia}+\sum P_{ia}C_{ja}-{CFR}_{j} \right),$$

while $\delta$ can be calculated as

$$\delta=0.5\left( {CFR}_{i}-\sum P_{ia}C_{ja}+\sum P_{ja}C_{ia}-{CFR}_{j} \right).$$

Note that the age groups for group $i$ and group $j$ need to be the same. If this is not the case in the raw data and, for instance, one country reports counts in 5-year age groups (0-4, 5-9, 10-14, 15-19, …) and the other uses 10-year age groups (0-9, 10-19, …), then either the more finely grained data needs to be aggregated to match the coarser data, or the coarser data needs to be adjusted. We choose the latter approach (see appendix C below).

The intuition behind the formulas is as follows. The first two terms in brackets in the equation for $\alpha$ are ${CFR}_{i}-\sum P_{ja}C_{ia}$, or, replacing ${CFR}_{i}$ with its definition, $\sum P_{ia}C_{ia}-\sum P_{ja}C_{ia}$. The second sum in this expression captures how high the CFR would have been if group $i$ had the same age distribution of infections as group $j$. The difference to the actual CFR (the whole expression) then captures to what extent the CFR is higher than this hypothetical CFR because of the actually observed age distribution of detected infections. The third and the fourth term in brackets in the equation for $\alpha$ are following a similar logic, but using a different hypothetical comparison, asking how much the CFR of group $j$ would differ if the detected cases had the age distribution of group $i$. The formula for $\delta$ again follows a similar logic, but now replacing the age-specific CFRs instead of the age distribution. In summary, to decompose the difference between two CFRs requires nothing more than the two CFRs themselves as well as a few additional hypothetical CFRs.

To calculate the proportion $\alpha$ and $\delta$ contribute to the total difference one can use $\frac{|\alpha|}{|\alpha|+|\delta|}$ in case of $\alpha$ and $\frac{|\delta|}{|\alpha|+|\delta|}$ for the contribution of $\delta$.

## A.2 Interpretation

As an artificial example, assume that the CFR in country A is equal to 2 percent, while it equals 4 percent in country B. Subtracting the CFR of country A from country B gives a difference of 2 percentage points. If a large part of this difference is due to the age structure, then $\alpha$ could be $0.015$ and $\delta$ could be $0.005$. These sum to $0.02$, or 2 percentage points. If, as another example, two countries have the same age structure of cases, then $\alpha$ will be zero. A similar reasoning holds for $\delta$ if age-specific CFRs are the same for both countries being compared. In relative terms, the $\alpha$-component explains 75 percent of the difference between countries, while the $\delta$-component only explains 25 percent.

The total difference between two CFRs as well as both $\alpha$ and $\delta$ can be negative. The formulas for the relative contributions take this into account by using absolute values. If the total difference is positive and either $\alpha$ or $\delta$ are negative, it means that the corresponding part of the difference actually reduces the difference between CFRs. For instance, when comparing the CFR for one country at two points in time, the total difference could be $0.03$; i.e., the CFR increased by three percentage points. If in this case $\alpha$ would be negative, say $-0.01$, it would mean that the age distribution of cases over time became more favorable. $\delta$ would be $0.04$ in this scenario, and without changes in the age distribution of infections as captured through $\alpha$, the difference between CFRs would even have increased by four percentage points.

# B. Additional results

# B.1 Country comparisons

**Table S1: Results of the cross-country decompositions using Germany as a reference case.**

| **Country**  **(1)** | **CFR**  **(2)** | **Difference**  **(3)** | **Age (**$\alpha$**) component**  **(4)** | **Fatality (**$\delta$**) component**  **(5)** | **Age (**$\alpha$**) component, relative**  **(6)** | **Fatality (**$\delta$**) component, relative**  **(7)** |
| --- | --- | --- | --- | --- | --- | --- |
| South Korea | 0.022 | 0.024 | 0.018 | 0.006 | 74.7% | 25.3% |
| China | 0.023 | 0.024 | 0.011 | 0.012 | 47.2% | 52.8% |
| Germany | 0.046 |  |  | (Reference) |  |  |
| USA | 0.048 | -0.001 | 0.008 | -0.009 | 46.6% | 53.4% |
| New York City | 0.087 | -0.041 | 0.008 | -0.049 | 13.9% | 86.1% |
| Spain | 0.122 | -0.075 | -0.051 | -0.024 | 68.0% | 32.0% |
| Italy | 0.140 | -0.094 | -0.059 | -0.035 | 62.6% | 37.4% |

**TableS 2: Results of the cross-country decompositions using Italy as a reference case**

| **Country**  **(1)** | **CFR**  **(2)** | **Difference**  **(3)** | **Age (**$\alpha$**) component**  **(4)** | **Fatality (**$\delta$**) component**  **(5)** | **Age (**$\alpha$**) component, relative**  **(6)** | **Fatality (**$\delta$**) component, relative**  **(7)** |
| --- | --- | --- | --- | --- | --- | --- |
| South Korea | 0.022 | 0.118 | 0.077 | 0.041 | 65.3% | 34.7% |
| China | 0.023 | 0.117 | 0.057 | 0.060 | 48.7% | 51.3% |
| Germany | 0.046 | 0.094 | 0.059 | 0.035 | 62.6% | 37.4% |
| USA | 0.048 | 0.093 | 0.069 | 0.023 | 74.7% | 25.3% |
| New York City | 0.087 | 0.053 | 0.084 | -0.031 | 73.2% | 26.8% |
| Spain | 0.122 | 0.018 | 0.006 | 0.013 | 31.9% | 68.1% |
| Italy | 0.140 |  |  | (Reference) |  |  |

# B.2 Trends over time

**Table S3: Development of the case-fatality rate (CFR) over time in New York City.**

| **Date**  **(1)** | **CFR**  **(2)** | **Difference**  **(3)** | **Age (**$\alpha$**) component**  **(4)** | **Fatality (**$\delta$**) component**  **(5)** | **Age (**$\alpha$**) component, relative**  **(6)** | **Fatality (**$\delta$**) component, relative**  **(7)** |
| --- | --- | --- | --- | --- | --- | --- |
| March 22, 2020 | 0.009 |  |  | (Reference) | |  |
| April 5, 2020 | 0.038 | 0.029 | 0.004 | 0.025 | 12.4% | 87.6% |
| April 19, 2020 | 0.069 | 0.060 | 0.007 | 0.052 | 12.6% | 87.4% |
| May 3, 2020 | 0.079 | 0.070 | 0.008 | 0.062 | 11.8% | 88.2% |
| May 18, 2020 | 0.084 | 0.074 | 0.009 | 0.065 | 12.2% | 87.8% |
| June 1, 2020 | 0.084 | 0.075 | 0.009 | 0.066 | 12.0% | 88.0% |
| June 17, 2020 | 0.084 | 0.075 | 0.009 | 0.066 | 12.0% | 88.0% |
| June 30, 2020 | 0.087 | 0.078 | 0.009 | 0.069 | 11.7% | 88.3% |

**Table S4: Development of the case-fatality rate (CFR) over time in Spain.**

| **Date**  **(1)** | **CFR**  **(2)** | **Difference**  **(3)** | **Age (**$\alpha$**) component**  **(4)** | **Fatality (**$\delta$**) component**  **(5)** | **Age (**$\alpha$**) component, relative**  **(6)** | **Fatality (**$\delta$**) component, relative**  **(7)** |
| --- | --- | --- | --- | --- | --- | --- |
| March 21, 2020 | 0.060 |  |  | (Reference) | |  |
| March 29, 2020 | 0.086 | 0.026 | -0.001 | 0.027 | 3.0% | 97.0% |
| April 6, 2020 | 0.098 | 0.038 | 0.003 | 0.035 | 7.1% | 92.9% |
| April 15, 2020 | 0.105 | 0.044 | 0.012 | 0.032 | 27.1% | 72.9% |
| April 23, 2020 | 0.111 | 0.051 | 0.014 | 0.036 | 28.4% | 71.6% |
| May 1, 2020 | 0.116 | 0.056 | 0.018 | 0.037 | 32.7% | 67.3% |
| May 9, 2020 | 0.119 | 0.058 | 0.019 | 0.039 | 32.8% | 67.2% |
| May 21, 2020 | 0.122 | 0.062 | 0.020 | 0.042 | 31.8% | 68.2% |

**Table S5: Development of the case-fatality rate (CFR) over time in Germany.**

| **Date**  **(1)** | **CFR**  **(2)** | **Difference**  **(3)** | **Age (**$\alpha$**) component**  **(4)** | **Fatality (**$\delta$**) component**  **(5)** | **Age (**$\alpha$**) component, relative**  **(6)** | **Fatality (**$\delta$**) component, relative**  **(7)** |
| --- | --- | --- | --- | --- | --- | --- |
| March 21, 2020 | 0.018 |  |  | (Reference) | |  |
| April 5, 2020 | 0.042 | 0.025 | 0.020 | 0.004 | 82.5% | 17.5% |
| April 19, 2020 | 0.050 | 0.033 | 0.029 | 0.003 | 89.7% | 10.3% |
| May 3, 2020 | 0.051 | 0.033 | 0.031 | 0.002 | 93.5% | 6.5% |
| May 18, 2020 | 0.050 | 0.032 | 0.031 | 0.001 | 95.5% | 4.5% |
| June 1, 2020 | 0.049 | 0.031 | 0.030 | 0.001 | 96.9% | 3.1% |
| June 17, 2020 | 0.048 | 0.030 | 0.029 | 0.001 | 97.9% | 2.1% |
| June 30, 2020 | 0.046 | 0.029 | 0.028 | 0.000 | 98.8% | 1.2% |

# B.3 Decomposition results based on estimates of excess mortality

Here we explore the application of our method to estimates of excess mortality. Estimates of excess mortality might be an alternative to deaths attributed to COVID-19, as the latter are prone to underreporting and misclassification. For these estimates, we used the Short-term Mortality Fluctuations (STMF) data series provided by the Human Mortality Database (HMD) [2]. Most countries report data in 5-year age groups, and when wider age groups were provided (e.g., Germany and the United States) we applied the PCLM method [3] to break down the data into the same age intervals. To estimate excess mortality by age, we use the methodology proposed by the EuroMOMO project [4]. Specifically, for each country, we calculate a Poisson regression accounting for secular and seasonal changes in mortality. We fit the model to weekly age-specific death counts for up to five years previous to 2020, excluding winter (weeks 46-14) and summer (weeks 27-35) periods to avoid period mortality disturbances, such as those caused by influenza and heatwaves. The precise number of years depends on the country; for instance, for Germany data is only available from 2016 on. We predict weekly death counts for 2020 based on the Poisson model. Age-specific excess mortality is then calculated as the cumulative number of observed deaths up to a certain week minus the predicted cumulative number of deaths for the same age group and week. If the resulting number is negative, we set it to zero. These estimates are then used in the calculation of CFRs instead of deaths attributed to COVID-19. Confirmed COVID-19 cases are still used as denominators for the CFRs. In some instances, this results in age-specific CFRs above one. These are set to one.

Results are shown in Table S6 using Germany as a reference country and decomposing the difference to Italy, the United States, and Spain. China, South Korea, and New York City are not included, as the (HMD) does not provide weekly mortality data. All results are for early-May or mid-May, as more recent data on weekly mortality is not available yet. Compared to the findings in the main text in Table 2, CFRs are higher, and in the cases of Spain and Italy considerably higher. The higher CFR in Italy and Spain compared to Germany is partly due to the age distribution, partly due to the fatality of COVID-19. The same goes for the difference for the United States, which has a slightly lower CFR than Germany.

**Table S6: Results of the cross-country decompositions of case-fatality rates (CFRs) based on excess mortality.**

| **Country (1)** | **Week (2)** | **CFR (3)** | **Difference**  **(4)** | **Age (**$\alpha$**) component**  **(5)** | **Fatality (**$\delta$**) component**  **(6)** | **Age (**$\alpha$**) component, relative**  **(7)** | **Fatality (**$\delta$**) component, relative**  **(8)** |
| --- | --- | --- | --- | --- | --- | --- | --- |
| USA | 21 | 0.066 | 0.001 | 0.004 | -0.003 | 56.6% | 43.4% |
| Germany | 19 | 0.067 |  | (Reference) |  |  |  |
| Spain | 19 | 0.218 | -0.151 | -0.077 | -0.075 | 50.7% | 49.3% |
| Italy | 19 | 0.225 | -0.158 | -0.089 | -0.070 | 56.1% | 43.9% |

These results need to be interpreted with care for several reasons. Our estimates of excess mortality are likely biased. The main issue is that, essentially, excess mortality estimates compare death counts for 2020 which are affected by both the pandemic and measures such as lockdowns with death counts from previous years not affected by major epidemics and without major measures affecting mortality. “True” excess mortality estimates would require a comparison in which only the epidemic status changes, and not the counter-measures. This is because extraordinary lockdown measures also affect causes of death other than COVID-19, and they may have both reduced mortality from accidents and violent causes - mainly at young ages - and increased mortality due to avoided hospital care or delayed treatment for other conditions, mainly at old ages. Moreover, we relate the estimates of excess mortality to confirmed cases, the latter most likely underestimating the true number of cases considerably [5]. These problems are also evident in excess mortality estimates below zero and age-specific CFR estimates above one, as mentioned above.

# C. Additional information on data: Age groups

The data we use is provided in different age groups, depending on the country. The following age groups are used in the original data for both case counts and death counts:

• China: 0-9, 10-19, 20-29, 30-39, 40-49, 50-59, 69-69, 70-79, 80+

• Germany: 0-4, 5-14, 15-34, 35-59, 60-79, 80+

• Italy: 0-9, 10-19, 20-29, 30-39, 40-49, 50-59, 69-69, 70-79, 80-89, 90+

• Spain: 0-9, 10-19, 20-29, 30-39, 40-49, 50-59, 69-69, 70-79, 80+

• South Korea: 0-9, 10-19, 20-29, 30-39, 40-49, 50-59, 69-69, 70-79, 80+

• United States: 0-19, 20-44, 45-54, 55-64, 65-74, 75-84, 85+

• United States (New York City): 0-18, 18-44, 45-64, 65-74, 75+

For the decomposition, the age groups have to match. This is the case for China, Spain, South Korea, and Italy; in the case of the latter the age categories 80-89 and 90+ have to be merged. The age groups provided for Germany and the United States are problematic, as they do not match the age groups of any other country. Aggregating the age groups, as for Italy, does not help, either. For instance, the age category of 20 years to 44 years available for the US cannot be created based on the German data. To deal with this issue, the database project from which we obtained the case and death counts [6] uses a smoothing approach to estimate counts for age groups 0-9, 10-19, …, 80+ [3].

# D. Excel spreadsheet and R code

All R code used to produce the results in the paper as well as the data can be downloaded from <https://osf.io/vdgwt/>. An Excel spreadsheet containing several examples can be found in the same repository.

# References

[1] Kitagawa, EM. Components of a difference between two rates. *Journal of the American Statistical Association* 1955; 50:1168–1194. doi: 10.2307/2281213

[2] Human Mortality Database. University of California, Berkeley (USA), and Max Planck Institute for Demographic Research (Germany). Available at [www.mortality.org](http://www.mortality.org) or [www.humanmortality.de](http://www.humanmortality.de) (data downloaded on July 19th 2020).

[3] Rizzi, S., Gampe, J., Eilers, P. H. C. Efficient Estimation of Smooth Distributions From Coarsely Grouped Data. *American Journal of Epidemiology* 2015; 182: 138-147. [doi: 10.1093/aje/kwv020](https://doi.org/10.1093/aje/kwv020)

[4] Gergonne B, Mazick A, O’Donnell J, Oza A , Cox B, Wuillaume F et al. A European algorithm for a common monitoring of mortality across Europe. euroMOMO Work package 7 report. 2020. https://www.euromomo.eu/uploads/pdf/wp7_report.pdf

[5] Bohk Ewald C, Dudel C, Myrskylä M. A demographic scaling model for estimating the total number of COVID-19 infections. medRxiv preprint 2020. doi: 10.1101/2020.04.23.20077719

[6] Riffe T, Acosta E, Aburto JM, Alburez-Gutierrez D, Altová A, Baselini U et al. Database on age-specific COVID-19 stuff. COVID-19 cases and deaths by age Database (CAgeDB). 2020. https://osf.io/mpwjq/, doi: 10.17605/OSF.IO/MPWJQ.
