# Supplementary material for: Monitoring trends and differences in COVID-19 case-fatality rates using decomposition methods: Contributions of age structure and age-specific fatality
Source: PLoS One. 2020 Sep 10;15(9):e0238904. doi: 10.1371/journal.pone.0238904 (PMC7482960; doi:10.1371/journal.pone.0238904)
Supplement: Attachment — Submitted filename: Response to reviewers.docx [file pone.0238904.s002.docx]

Dear Prof. Masquelier,

We are pleased to resubmit a revised version of our paper “Monitoring trends and differences in COVID-19 case-fatality rates using decomposition methods: Contributions of age structure and age-specific fatality” (Manuscript ID PONE-D-20-12360). We would like to thank the reviewers for their valuable comments, which helped to improve the paper considerably. Please find below our replies to their suggestions.

The most substantial revisions concern the inclusion of a new analysis decomposing differences between countries in age-specific excess mortality. We also added results on trends over time covering three additional countries (Germany, Spain, the U.S.). Moreover, we updated all results based on recent data, including the Excel spreadsheet which demonstrates our calculations, and we added new references. Please note that in the highlighted version of the manuscript we did not highlight updates of results already included in the previous version, as this would have meant highlighting large parts of the text and of all tables.

We also implemented several other changes in the manuscript, such as an update of the paragraph on seroprevalence studies in the Discussion section, and we updated the R code to make it easier to update our results in the future. We tried to accommodate all edits without increasing the length of the paper too much.

# Reviewer 1 comments

**Comment 1**: One point of clarification: Could the authors clarify in the text if you also use data over time for Germany as in Figure 1.

**Reply**: Thank you for your positive assessment of our paper. We now added an analysis over time for Germany, which is included in the supplementary materials, and we mention this in the abstract (p. 2) as well as in the introduction (p.4, 3^rd^ paragraph; also see our reply to comment 2 of Reviewer 2 below).

# Reviewer 2 comments

**Comment 1**: p.15, first paragraph: The authors mention that the method could be used on excess all-cause weekly mortality counts. This data is now available for some countries (see the Human Mortality Database for example). I would suggest that the authors use excess deaths that are less subject to bias and compare their results.

**Reply**: Thank you for your very helpful comments. We calculated excess mortality estimates by age from the Short-term Mortality Fluctuations (STMF) data series provided by the Human Mortality Database (HMD) using the methodology of the EuroMOMO project, and we present decomposition results for these in the supplementary materials (p. 5-6 in the supplementary materials). We refer to this in the main text on p. 16, paragraph 2. As it turns out that excess mortality estimates are not without problems, at least for a decomposition analysis, we also mention this in the main text (p. 16, paragraph 2). The main issue is that, essentially, excess mortality estimates compare death counts for 2020 which are affected by both the pandemic and measures such as lockdowns with death counts from previous years which were not affected by major epidemics and without major measures affecting mortality. “True” excess mortality estimates would require a comparison in which only the epidemic status changes, and not the counter-measures. This means that standard excess mortality estimates are potentially biased downward. This can be clearly seen for some age groups for which estimates of excess mortality are below the number of confirmed deaths. There are also a few instances where the number of excess deaths is above the number of confirmed cases, leading to CFRs above one. Decomposition results based on such excess mortality estimates are thus hard to interpret, which is why we only present them in the supplementary materials.

**Comment 2**: p.10, Trends over time were only studied for Italy. I would suggest the authors to analyze how trends differ between countries (i.e with Spain, the US) or at least address why they don’t.

**Reply**: We expanded the analysis of trends over time to three additional countries or regions: Germany, Spain, and New York City. The results are included in the supplementary materials (Tables S3, S4, and S5 in the supplementary materials), and discussed in the main text on p. 12, 1^st^ paragraph. These additional results show that trends over time can also be driven by the age component, which largely contributed to the increase in the CFR in Germany (p 12, 1^st^ paragraph).

**Comment 3**: The authors should present data on the amount of testing performed by each country, if possible, over the available age groups (and over time for Italy). It would be more transparent to read results having this information on the side.

**Reply**: We added numbers on tests in Table 1 (p. 6).

**Comment 4**: p.4, “Counts of confirmed cases and deaths might not be comparable across countries because of differences in case and death definitions”. Despite rightly highlighting this fact, the purpose of the selected method is cross-country comparisons. Having this in mind, can the results support any conclusion?

**Reply**: The method can be used for cross-country comparisons as well as for the analysis of trends over time. In the latter case, comparability of the case and death definitions over time will often be the case. For cross-country comparisons, comparability is more problematic, and it motivated the exclusion of France from the analysis, which we had included in earlier drafts, as deaths at home were not captured in the French data in contrast to many other countries. For the countries we include in our analysis there might be differences in definitions; however, these are small, and we are not aware of differences that would render them completely incomparable.

**Comment 5**: Amount of missing data is only presented for Spain. What is the amount of missing data in the other countries? Could you add this information for all the countries?

**Reply**: We added this information to the text in paragraph 2, p. 7. For the Chinese and the Korean data, all ages are known. For the Italian data, the age of all deaths is known, and missing for 38 cases. For Germany and New York City the number of cases and deaths with missing age is also rather small (less than 0.5 percent of all cases/deaths). For the U.S., the number of cases with missing age is relatively large and amounts to roughly 15% of all cases. However, for most deaths in the U.S. data the age is known (less than 0.5 percent missing).

**Comment 6**: p.6, “The database project imputed the missing age using the observed age distribution of cases or deaths, respectively”. Doesn’t this method exacerbate the bias if some ages are rarely reported (i.e deaths in nurse houses)?

**Reply**: As for most countries the number of cases or deaths without age is rather low or zero we do believe that this is not a cause for concern. The only exceptions are the United States and Spain, and we now state more clearly that this could bias the results (p. 7, 2^nd^ paragraph).

**Comment 7**: p.7, “Counts were split using a recently proposed method tailored for this data situation [9,10]”. For transparency, I would recommend to highlight assumptions required by this method (such as the underlying smoothness assumption of the counts).

**Reply**: We added the smoothness assumption to the text (p. 7, paragraph 3). We use offsets for splitting, which means that the smoothness assumption shifts from counts to rates.

**Comment 8**: In order to provide more descriptive statistics, could figure 1 be done for all the countries included in the analysis?

**Reply**: We extended the number of countries included in Figure 1. However, as not to lead to confusion, we only included countries for which we conduct an analysis of the trend over time (see comment 1 of Reviewer 1). Besides, the comparatively low death counts of South Korea and high case counts of the US makes it difficult to include them in the same figure as the other countries.
